# Myeloid phenotypes in severe COVID-19 predict secondary infection and mortality: a pilot study

**DOI:** 10.1186/s13613-021-00896-4

**Published:** 2021-07-14

**Authors:** Clémence Marais, Caroline Claude, Nada Semaan, Ramy Charbel, Simon Barreault, Brendan Travert, Jean-Eudes Piloquet, Zoé Demailly, Luc Morin, Zied Merchaoui, Jean-Louis Teboul, Philippe Durand, Jordi Miatello, Pierre Tissières, for The COVID-19 Immune Suppression (CLOVIS) Study Group, Simon Barreault, Mélissa Beggaz, Ramy Charbel, Caroline Claude, Zoé Demailly, Philippe Durand, Gaspard Gerschenfeld, Jessica Giraldi, Matteo Guerra, Manon Hily, Martin Journaux, Christopher Lai, Pauline Leroux, Clémence Marais, Zied Merchaoui, Jordi Miatello, Clarisse Niçaise, Jean-Eudes Piloquet, Melissa Ren, Marie Simbozel, Nada Semaan, Jean-Louis Teboul, Pierre Tissieres, Brendan Travert

**Affiliations:** 1 Pediatric « Adult COVID-19-Converted » Intensive Care and Neonatal Medicine, AP-HP Paris Saclay University, Bicêtre Hospital, 78, Rue du Général Leclerc, 94275 Le Kremlin-Bicêtre, France; 2 Institute of Integrative Biology of the Cell, CNRS, CEA, Univ. Paris Saclay, Gif-sur-Yvette, France; 3 Pediatric Intensive Care, Nantes University Hospital, Nantes, France; 4 Medical Intensive Care, AP-HP Paris Saclay University, Bicêtre Hospital, Le Kremlin-Bicêtre, France; 5 FHU SEPSIS, AP-HP/Université Paris Saclay/Inserm, Le Kremlin-Bicêtre, France

**Keywords:** COVID-19, Sepsis-induced immunodepression, Secondary infection, mHLA-DR, Myeloid-derived suppressor cells

## Abstract

**Background:**

De-regulated host response to severe coronavirus disease 2019 (COVID-19), directly referring to the concept of sepsis-associated immunological dysregulation, seems to be a strong signature of severe COVID-19. Myeloid cells phenotyping is well recognized to diagnose critical illness-induced immunodepression in sepsis and has not been well characterized in COVID-19. The aim of this study is to review phenotypic characteristics of myeloid cells and evaluate their relations with the occurrence of secondary infection and mortality in patients with COVID-19 admitted in an intensive care unit.

**Methods:**

Retrospective analysis of the circulating myeloid cells phenotypes of adult COVID-19 critically ill patients. Phenotyping circulating immune cells was performed by flow cytometry daily for routine analysis and twice weekly for lymphocytes and monocytes subpopulations analysis, as well as monocyte human leukocyte antigen (mHLA)-DR expression.

**Results:**

Out of the 29 critically ill adult patients with severe COVID-19 analyzed, 12 (41.4%) developed secondary infection and six patients died during their stay. Monocyte HLA-DR kinetics was significantly different between patients developing secondary infection and those without, respectively, at day 5–7 and 8–10 following admission. The monocytes myeloid-derived suppressor cells to total monocytes ratio was associated with 28- and 60-day mortality. Those myeloid characteristics suggest three phenotypes: hyperactivated monocyte/macrophage is significantly associated with mortality, whereas persistent immunodepression is associated with secondary infection occurrence compared to transient immunodepression.

**Conclusions:**

Myeloid phenotypes of critically ill COVID-19 patients may be associated with development of secondary infection, 28- and 60-day mortality.

**Supplementary Information:**

The online version contains supplementary material available at 10.1186/s13613-021-00896-4.

## Introduction

Critically ill patients with COVID-19 are now recognized to develop frequently secondary bacterial infection with an incidence ranging between 14 and 25% [1, 2]. Among the contributing factors for secondary infections, prolonged ICU stay is well established, but environmental conditions are known to greatly influence its development. The host response to acute and/or prolonged aggression is well recognized to participate in critical illness-induced immunodepression and its clinical consequences [3]. Both innate and adaptive immune signatures characterize this condition, but myeloid function and phenotypic profiles are the most robust predictors of acquisition of secondary infections and mortality [4].

Monocytes have the capacity to detect aggression and danger signals, to trigger inflammation and to initiate resolution of inflammation. This extreme functional plasticity is closely associated with myeloid phenotypes throughout myelopoiesis and maturational process modifications. Various inflammatory/infectious signals (e.g., calprotectin, Interleukin (IL)-6, Toll Like Receptors (TLR) agonists) are identified to reorient and promote release from the bone marrow of immature myeloid cells that suppress both innate and adaptive immune response [5]. Those cells, named myeloid-derived suppressor cells (MDSC), are now well identified to participate in the host response to infection by blocking T lymphocyte and natural killer cells proliferation, and production of high amount of immunosuppressive cytokine such as IL-10. In addition, the resulting pro-/anti-inflammatory function of monocytes is globally reflected by the level of surface expression of human leukocyte antigen (HLA)-DR [6] which is a highly predictive of secondary infection development and mortality in patients with sepsis [7, 8]. Increasing evidence suggests a central role of myeloid cells and of severe immunosuppression in the pathogenesis of severe COVID-19 [9–11].

Hereby, we aimed at defining the myeloid cells phenotypes during the first 2 weeks following ICU admission of critically ill COVID-19 and explore their relations with development of secondary infections, 28- and 60-day mortality.

## Methods

All critically ill adult patients with confirmed COVID-19 (positive RT-PCR for SARS-CoV-2 and suggestive chest CT-scan) admitted to the Pediatric ICU of the Bicêtre Hospital, AP-HP Paris Saclay University, between March and April 2020 were included. Due to the shortage of adult ICU beds, 16 PICU beds were converted to admit adult COVID-19 critically ill patients and staffed with the PICU professionals reinforced by professionals coming from preserved French regions [12]. A senior adult intensivist (JLT) completed the team. All patients were prospectively included in the CLOVIS cohort study (ClinicalTrial.gov Identifier: NCT04544878). The study (French study classification: MR004) was approved by the local IRB and French Data Protection Authority (CNIL registration number: 2219981) waiving the need of written consent. All patients or relatives received information on the study and could refuse to participate at any time. Clinical characteristics including age, gender, comorbidities, occurrence of organ failure, infective complications and related microbiological documentation, length of stay, 28- and 60-day mortality were obtained. Biological characteristics included on admission ferritin, brain natriuretic peptide, troponin T, complement factors, immunoglobulins and sub-classes, daily standard blood workup, and twice weekly non-specific inflammatory markers (e.g., C-reactive protein, procalcitonin), circulating cell phenotyping (complete cell count, T cells and subtypes, B cells, natural killer cells, monocytes and subtypes, neutrophils and subtypes) and monocyte histocompatibility leukocyte antigen (HLA)-DR expression measurement (anti-HLA-DR/anti-monocyte Quantibrite assay, BD Biosciences, San Jose, USA). Data were reported at admission, and at four periods within the first 2 weeks: days 1–4, days 5–7, days 8–10 and days 11–14. Circulating cell phenotyping was performed using ethylenediaminetetraacetic acid (EDTA) anticoagulated blood. Detailed antibodies used for myeloid phenotyping are reported elsewhere (see Additional file 1: Table S1). Monocyte subsets analysis was based on HLA-DR and CD11b expression in CD19^−^CD14^+^CD15^−^ cells. Among these, monocytes myeloid-derived suppressor cells (M-MDSC) were identified as CD11b^+^ HLA-DR^−^. Thresholds selected for HLA-DR “low-immunosuppressed” group was set at < 15,000 antibody per cell (AB/C) and “high-elevated” group > 30,000 AB/C according to recent published data in COVID-19 critically ill patients [11]. Standard immunophenotyping was performed for lymphocytes T and subclass, NK cells, lymphocytes B, neutrophils. The primary endpoint was the occurrence of secondary infection during the ICU stay as defined by IDSA definitions [13, 14]. The secondary endpoint was 28- and 60-day mortality.

Data are described as number (%) and median (interquartile range (IQR) for categorical and continuous variables, respectively). Comparison of baseline characteristics between patients with a blood sample was done using the Fisher’s exact test or the Chi-square test, as appropriate. The Wilcoxon Mann–Whitney *U* test was used to determine differences between immunological variables at each time point. We measured the discrimination of M-MDSC to total monocytes ratio using the area under the receiver operating characteristics (AUROC) curve. The best threshold was obtained with the calculation of sensitivity, specificity, positive and negative predictive values and the Youden’s index (*Y* = sensitivity + specificity − 1). A *p*-value of less than 0.05 was considered statistically significant. All analyses were performed using GraphPad Prism, v 8.4.2 (GraphPad Software, LLC).

## Results

**Fig. 1:**
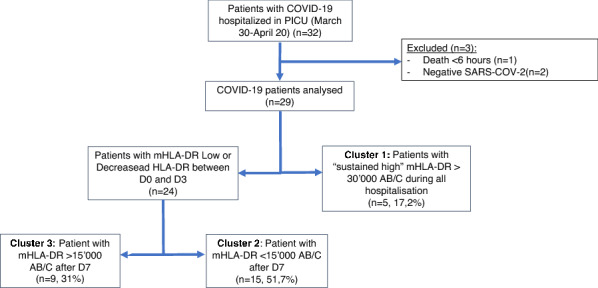
Flowchart

From March 26 to April 15, 2020, 32 critically ill adult patients with suspected COVID-19 were admitted to the Pediatric ICU of the Bicêtre Hospital, AP-HP Paris Saclay University. Out of the 32 patients, one died within the first 6 h following admission and two ultimately had negative SARS-Cov-2 PCR and were definitively considered as non-COVID-19 patients (Fig. 1). Twenty-nine patients were analyzed and their characteristics displayed in Table 1. Secondary infection occurred in 12 patients (41.4%) and six patients (20.7%) died in the ICU. Length of stay in the ICU ranged between 7 and 28 days. At the end of the ICU stay, all survivors were transferred to an adult stepdown unit or wards of our hospital. Five patients were transferred to a medical unit with ventilator rehabilitation facilities. Secondary infections were primarily ventilator-associated pneumonia including pulmonary abscess (*n* = 2) and *Aspergillus* spp pneumonia (*n* = 2) (see Additional file 1: Table S2). On univariate analysis, age, SAPS II, comorbidities and mechanical ventilation were significantly different between patients developing or not secondary infection (Table 1). No biologic markers but NK cells count, monocytes count and M-MDSC to total monocytes ratio significantly differed between both groups.

**Table 1:** Patients characteristics

	No secondary infection (*n* = 17)	Secondary infection (*n* = 12)	*p* value
Age (years)	52 (47–54)	64 (62–66)	0.005
BMI (kg/m^2^)	28.5 (26.3–31.4)	29.3 (27.4–33)	0.44
Gender male	10 (58.8)	10 (83.3)	0.16
SOFA score			
Day 1	7 (4–11)	10 (7–14)	0.095
Day 3	7 (4–10)	11 (7–14.5)	0.024
Day 7	6 (4.5–7)	11 (8–14.5)	0.013
Day 10	3 (3–6)	11 (9–13.5)	0.02
SAPS II score			
Day 1	45 (27–58)	59 (54–67)	0.03
Day 3	48 (24–55)	62 (58–64)	0.04
Day 7	40 (25–49)	60(58–69)	0.02
Day 10	23 (13–35)	65 (56–70)	0.001
Comorbidities			
Cardiologic	5 (29.4)	10 (83.3)	0.004
Hypertension	5 (100)	9 (90)	–
Hypertrophic cardiomyopathy	1 (20)	0	–
Peripheral artery occlusive disease	1 (20)	1 (10)	–
Ischemic cardiopathy	0	2 (20)	–
Pneumologic	2 (11.8)	1(8.3)	0.77
Neurologic	2 (11.8)	3 (25)	0.35
Immunologic	3 (17.6)	1 (8.3)	0.47
Metabolic	7 (41.2)	8 (66.7)	0.18
Supportive care			
Norepinephrine	5 (29.4)	5 (41.7)	0.49
Mechanical ventilation	12 (70.6)	12 (100)	0.04
CRRT	3 (17.6)	5 (41.7)	0.154
Immunotherapy	1 (5.8)	2 (33.3)	0.55
Laboratory values at admission			
Leucocytes (/mm^3^)	7520 (5970–9330)	11,910 (7160–14,750)	0.082
Neutrophils (/mm^3^)	6420 (4880–8310)	10,010 (6238–12,225)	0.09
Immature neutrophils (%)	4 (2.57–7.54)	4.9 (2.5–6.1)	0.98
Lymphocytes (/mm^3^)	800 (600–1191)	1235 (663–1442)	0.143
CD4 (/mm^3^)	330 (222.5–526.5)	333 (240.5–481.5)	0.917
CD8 (/mm^3^)	167 (114.5–257)	346 (163–399.5)	0.102
NK (/mm^3^)	74 (63.25 -109.25)	127 (95–249)	0.046
B (/mm^3^)	136 (97.5–279)	132 (100–333.5)	0.827
Monocytes (/mm^3^)^a^	198.7 (81.5–284)	328.6 (170.9–453.8)	0.03
mHLA-DR (AB/C)	17,737 (10,060–32,503)	11,877 (7039–22,702)	0.3
M-MDSC (%)	3.8 (0.95–13.96)	10.25 (4.14–29.32)	0.04
Immunoglobulin G (g/L)	9.87 (7.6–11.35)	10.8 (8.71–13.80)	0.364
Immunoglobulin M (g/L)	1.22 (0.7–1.39)	0.81 (0.63–1.30)	0.427
Immunoglobulin A (g/L)	2.73 (2.21–3.3)	2.49 (2.21–5.02)	0.584
C3 (g/L)	1.28 (1.05–1.43)	1.4 (1.15–1.45)	0.568
C4 (g/L)	0.27 (0.23–0.36)	0.29 (0.22–0.34)	0.765
CH50 (U/mL)	70 (56.5–73.3)	70 (57–73)	0.943
Platelets (× 10^3^/mm^3^)	271(182–340)	444(263–503.7)	0.097
Fibrinogen (mg/dL)	7.3 (6.5–8.1)	8.1 (7–8.3)	0.28
d-dimers (g/dL)	2145 (1100–3595)	2859 (1612–2870)	0.29
Ferritin (μg/L)	1223 (818.25–1378.5)	1691 (303–4406.5)	0.287
Creatinine (μmol/L)	67 (50–109)	88.5 (65.8–145)	0.25
Total bilirubin (μmol/L)	9 (6–12)	11 (5.75–21.5)	0.73
PCT (ng/mL)	0.64 (0.34–2.77)	0.71(0.36–3.22)	0.9
CRP (mg/L)	131.5 (89.7–210.5)	188(98.5–265)	0.34
Outcome			
28-day mortality	3 (17.6)	3 (25)	0.63
60-day mortality	5 (29.4%)	6 (50)	0.26
PICU length of stay	10 (7–15)	26 (21.5–28)	0.0001

Values are expressed as median (IQR) or number (%)

BMI, body mass index; SOFA, Sequential Organ Failure Assessment; SAPS, Simplified Acute Physiological Score; CRRT, continuous renal replacement therapy; CD, cluster of differentiation; mHLA-DR, monocyte histocompatibility leucocyte antigen-DR; M-MDSC, monocyte myeloid-derived suppressor cell (CD11b^+^ HLA-DR^−^), C, complement; CH50, total complement activity; PCT, procalcitonin; CRP, C-reactive protein; PICU, pediatric intensive care unit

^a^Total monocytes: CD19^−^ CD14^+^ CD15^−^, monocytes subtypes based on CD11b and HLA-DR

**Fig. 2:**
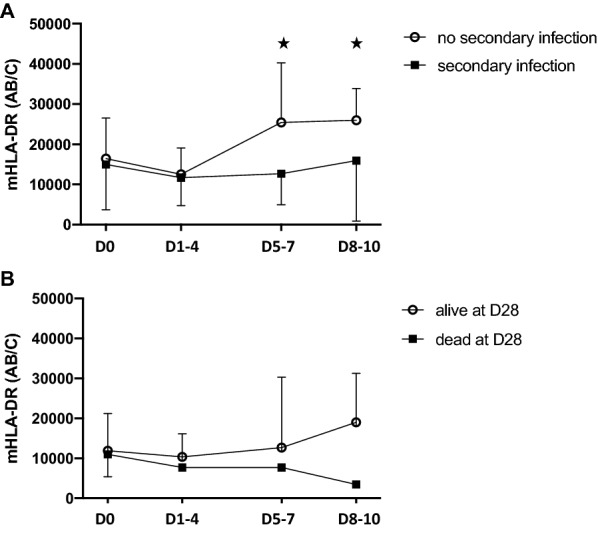
Monocyte HLA-DR kinetics. Monocyte HLA-DR antibody per cell surface expression from admission to day 8–10 in patients with secondary infection (**a**) or survivors at day 28 (**b**). *p < 0.05

**Fig. 3:**
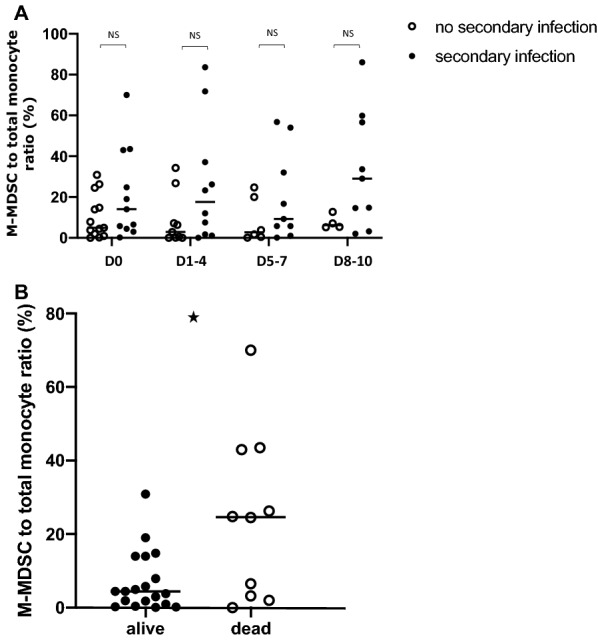
Monocyte myeloid-derived suppressor cells kinetics. Monocyte myeloid-derived suppressor cells (M-MDSC) kinetics from admission to day 8–10 in patients with secondary infection (**a**) or survivors at day 28 (**b**). **p < 0.005

Five patients had persistently high mHLA-DR throughout their ICU stay. In the remaining 24 patients, mHLA-DR at admission was similar, but patients with secondary infection had a persistently low mHLA-DR level throughout the first 2 weeks, whereas those without secondary infection significantly increased their mHLA-DR within the first 5–7 days after admission (median range increase from day 1–4: 14,826 to 25,355 AB/C at day 5–7 and 14,826 to 27,082 at day 8–10 AB/C) (Fig. 2a). Interestingly, no significant change in mHLA-DR occurs in both groups between days 5 to 10, suggesting that most of the myeloid response is set in the first days following admission (see Additional file 1: Figure S1). This significant deviation of mHLA-DR between patients developing or not secondary infection occurred 1 to 3 days before infection diagnosis was made. No significant difference in mHLA-DR was found between survivors and deceased ones during the study (Fig. 2b). No statistical difference in the percentage of M-MDSC to total circulating monocytes between patients with or without secondary infection was observed (Fig. 3a). M-MDSC proportion to total circulating monocytes was associated with 60-day survival (AUROC curve = 0.70, 95% CI 0.39 to 1) with M-MDSC to total monocytes being significantly lower at admission in patients alive at day 60 (*n* = 10/29, *p* = 0.044) (Fig. 3b). A cut-off value of 21% (Youden index = 0.54) was associated with a sensitivity of 67% and specificity of 87%.

**Fig. 4:**
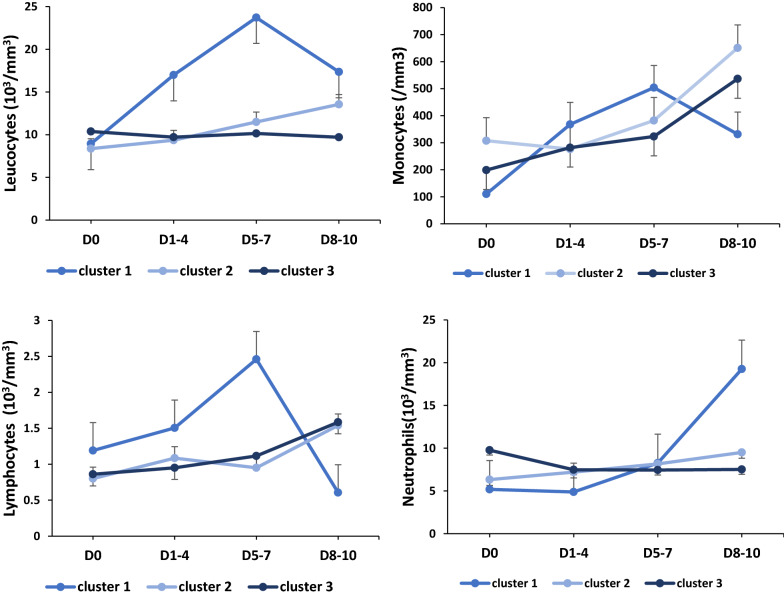
Circulating cells kinetics in severe COVID-19 myeloid phenotypes

Circulating myeloid cell phenotyping suggested three different groups of patients according to the evolution of the mHLA-DR rate: patients with persistently high mHLA-DR > 30,000 AB/C during the study (cluster 1), patients with persistently low mHLA-DR < 15,000 AB/C after day 5–7 (cluster 2) and patients with rising mHLA-DR > 15,000 AB/C after day 5–7 (cluster 3) (Table 2). On admission neither clinical nor biochemical parameters, but circulating cell phenotypes, differentiated the three phenotypes. Although on admission leukocytes count were similar (Fig. 4), patients from cluster 1 showed a non-significant trend toward early increased in leukocytes (Wilcoxon sign rank test, *p* = 0.06). Since patient from cluster 1 always displayed no to low M-MDSC (range: 0 to 3.19%), analysis of M-MDSC proportion to total circulating monocytes, without cluster 1 patients, was significantly associated with 28 days mortality (*p* = 0.003, AUROC curve = 0.94, 95% CI 0.84 to 1; cut-off value 18%, Youden Index = 0.85). The three phenotypes were associated with clinical outcomes: patients from cluster 1 displayed a hyperactivated monocytes/macrophage (HAMM) phenotype associated with the highest mortality, whereas those from cluster 2 and 3 displaying either prolonged immunodepression (PID), and transient immunodepression (TID) were differentially associated with occurrence of secondary infection (Table 3). Interestingly, the development of a secondary infection among the M-MDSC to total monocytes ratio > 18% group only occurred in patients with PID.

**Table 2:** Severe COVID-19 myeloid phenotypes at admission

	Cluster 1 (HAMM, *n* = 8)	Cluster 2 (PID, *n* = 18)	Cluster 3 (TID, *n* = 9)	*p*
Age (years)	44 (43–57)	63 (59–67)	55 (53–59)	0.04
BMI (kg/m^2^)	27.6 (26.3–31.5)	29.4 (26.7–31.8)	29.2 (26.4–31.4)	0.94
Gender male	3 (60%)	12 (66%)	5 (55%)	0.07
SOFA score	10 (2–14)	7 (6.5–12)	8 (6–12)	0.96
SAPS II score	57 (22–59)	55 (48–65)	54 (45–58)	0.63
Laboratory values				
Leucocytes (/mm^3^)	8940 (7520–15,600)	8370 (6800–11,260)	10,380 (5970–12,740)	0.74
Neutrophils (/mm^3^)	5180 (4770–8310)	6320 (5525–11,415)	9770 (7960–11,080)	0.66
Immature neutrophils (%)	7.3 (4–23)	3.3 (2.7–6.7)	4.6 (1.1–7.2)	0.39
Lymphocytes (/mm^3^)	1191 (970–1370)	800 (620–1340)	860 (780–1220)	0.87
CD4 (/mm^3^)	438 (308–670)	396 (230–529)	312 (226–339)	0.44
CD8 (/mm^3^)	246 (74–356)	268 (110–376)	213 (162–243)	0.93
NK (/mm^3^)	69 (63–92)	102 (65–167)	111 (91.5–193.5)	0.24
B (/mm^3^)	301 (42–313)	132 (122–283)	127 (89–201)	0.80
Monocytes (/mm^3^)^a^	110.2 (51.6–284)	307.4 (169.4–350.3)	198.7 (81.5–270.3)	0.2
mHLA-DR (AB/C)	46,340 (41,550–48,017)	10,060 (6329–16,281)	15,646 (10,873–28,568)	0.002
M-MDSC (%)	1.8 (0.4–1.87)	13.9 (5.4–27.8)	3 (0.9–14.8)	0.009
CD11b^+^ HLA-DR^+^	96.2 (95.8–97.95)	82.2 (69.0–94.6)	96.9 (85.2–14.8)	0.01
CD11^−^ HLA-DR^+^	0.37 (0–1.8)	0 (0–0.3)	0 (0–0)	0.28
LNR (%)	20.3 (11–26.4)	12.6 (9.2–17.4)	10.7 (9.8–17.1)	0.45
LMR (%)	9.1 (3.4–10.8)	3.5 (2.6–4.3)	3.1 (2.2–5.2)	0.2
Immunoglobulin G (g/L)	7.66 (7.54–10.10)	9.93 (8.02–12.5)	11.5 (9.80–14.30)	0.30
Immunoglobulin M (g/L)	1.21 (0.90–1.34)	0.81 (0.63–1.24)	1.3 (0.90–1.55)	0.36
Immunoglobulin A (g/L)	3.40 (1.94–4.32)	2.63 (2.21–3.71)	2.80(2.20–3.30)	0.99
C3 (g/L)	1.32 (1.09–1.53)	1.28 (1.09–1.43)	1.39 (1.23–1.44)	0.73
C4 (g/L)	0.25 (0.23–0.25)	0.24 (0.16–0.35)	0.29 (0.28–0.36)	0.40
CH50 (U/mL)	68 (63–69)	64 (52.5–73)	79 (72–83)	0.16
Platelets (× 10^3^/mm^3^)	340 (333–387)	308 (237–482)	242 (182–312)	0.73
Fibrinogen (mg/dL)	8.1 (6.5–8.1)	7.2 (6.5–8.1)	8.1 (6.7–8.3)	0.81
d-dimers (g/dL)	2875 (2027.5–3370)	1620 (1480–3510)	2610 (1880–4000)	0.86
Ferritin (μg/L)	507 (418–919)	1416 (768–1730)	1313 (729–1675)	0.30
Creatinine (μmol/L)	67 (42–172)	68 (57–121)	85 (60–109)	0.84
Total bilirubin (μmol/L)	5 (5–6)	11 (6–17)	10 (8–21)	0.22
PCT (ng/mL)	0.57 (0.47–2.95)	0.54 (0.19–1.74)	0.88 (0.64–2.77)	0.29
CRP (mg/L)	99 (74.5–117)	163 (85–229)	206.5 (149.8–233)	0.33
Outcomes				
28-day mortality	2 (40%)	2(13%)	2 (22%)	0.36
60-day mortality	2 (40%)	5 (33%)	3 (33%)	0.24
PICU length of stay	7 (6–10)	20 (11–24)	15 (11–23.5)	0.30

Values are expressed as median (IQR) or number (%)

BMI, body mass index; SOFA, Sequential Organ Failure Assessment; CD, cluster of differentiation; LNR, lymphocytes-to-neutrophils ratio; LMR, lymphocytes-to-monocytes ratio; mHLA-DR, monocyte histocompatibility leucocyte antigen-DR; M-MDSC, monocyte myeloid-derived suppressor cell (CD11b^+^HLA-DR^−^), C, complement; CH50, total complement activity; PCT, procalcitonin; CRP, C-reactive protein; PICU, pediatric intensive care unit

^a^Total monocytes: CD19^−^ CD14^+^ CD15^−^, monocytes subtypes based on CD11b and HLA-DR

**Table 3:** Myeloid phenotypes diagnostic criteria in severe COVID-19 and targeted investigation and therapy

	Hyper-activated monocytes/macrophage (HAMM)	Transient immune-depression (TID)	Persistent immune-depression (PID)
Dynamic diagnostic criteria			
M-MDSC day 0^a,b^	< 5%		
mHLA-DR day 0^a^	> 30,000 AB/C	< 30,000 AB/C	
mHLA-DR day 5–7^a^		> 15,000 AB/C	< 15,000 AB/C
Targeted investigation	Screen for MAS-like: myelogram		Track VAP
Targeted therapy	If MAS-like, consider immunotherapy (e.g. Anti-IL6)		Consider empiric antibiotics by day 5–7

M-MDSC, monocytes myeloid-derived suppressor cell to total monocytes ratio defined as CD19^−^ CD14^+^ CD15^−^CD11b^+^HLA-DR^−^ cells/CD19^−^ CD14^+^ CD15^−^ cells; mHLA-DR, monocytes Human leukocyte antigen-DR; AB/C, antibody per cell; MAS, macrophage activation syndrome; VAP, ventilator-associated pneumonia

^a^Following ICU admission

^b^M-MDSC > 18% is associated with 28-day mortality in PID and TID, with PID systematically associated with secondary infection

Although few patients were included in therapeutic institutional immunotherapy study [15], independently to the myeloid phenotypes, one patient with HAMM and confirmed macrophage activation was treated with compassionate anti-IL6 therapy (tocilizumab) and successfully weaned from ECMO within 48 h and fully recovered.

## Discussion

This pilot study identified two prototypic myeloid characteristics associated in critically ill COVID-19 adult patients with the occurrence of secondary infection and mortality. Monocyte HLA-DR kinetics and presence of M-MDSC, both illustrating myeloid antigen presentation and immune cell suppressive function, showed specific profiles in severe COVID-19.

In contrast to published series suggesting a systematic decrease or low (< 15,000AB/C) mHLA-DR in severe COVID-19 [11], we identified three distinct mHLA-DR kinetics, suggestive of three clusters of patients (Fig. 1). Analysis of mHLA-DR kinetics has proven to be an accurate characterization of the immunological adaptation during critical illnesses. Although in COVID-19, mHLA-DR level seems not as low as what is seen in septic shock (< 8000 AB/C), in our study, profile kinetics seen in cluster 2 (PID) and cluster 3 (TID) were differentially associated with the development of secondary infection, which is in line with published experience in sepsis [16]. A decrease in mHLA-DR in severe COVID-19 was shown in both adults and children [17–21]. Spinetti et al*.* showed that critically ill patients initially admitted in general wards and secondarily transferred to the ICU, had a higher mHLA-DR than those directly admitted to the ICU [17]. Similarly, Moratto et al*.* showed that COVID-19 hospitalized patients progressing to critical illness had lower mHLA-DR level [18]. In a cohort of 157 COVID-19 patients, Wang et al. showed that the 62 patients who died had a significantly decreased mHLA-DR after 10 days [19]. Interestingly, preliminary report in children suggested that mHLA-DR was strongly decreased in children with severe COVID-19 multisystem inflammatory syndrome in children, but could not evidence an association with mortality [20]. In our study, mHLA-DR kinetics between deceased and survivors were not significantly different, although this result could have been hampered by the limited follow-up period.

In contrast to published experience, we identified a third cluster of patients, with persistently high mHLA-DR, suggestive of protracted monocyte activation phenotype. This mHLA-DR kinetic, although less frequent, was also identified in one series [17]. In our cohort, those patients had severe systemic inflammation, high ferritin, elevated fibrinogen and lymphopenia and in one patient, confirmed macrophage activation on bone marrow analysis reinforcing a potentially “hyperactivated monocyte/macrophage” (HAMM) phenotype. Use of mHLA-DR to identify critically ill patients with macrophage activation syndrome was recently suggested and may prove to be an effective screening criterion in patients with severe COVID-19 and suspected HAMM mimicking macrophage activation syndrome [22]. This observation further completed earlier observations suggesting that COVID-19 may be associated with macrophage activation-like syndrome [23–25].

Although not unique, impairment of myeloid response is increasingly recognized in COVID-19. Relative disappearance of non-classical CD14^low^CD16^high^ monocytes, seems to be characteristics of SARS-CoV-2 infection, not seen in other viral infections, and may be associated with severity [9, 26]. As shown, a second characteristic seen in severe COVID-19 is the decrease in HLA-DR expression on CD14^high^ monocytes that could be associated with altered responsiveness of circulating monocyte to TLRs bacterial agonists [27]. There is currently significant arguments to suggest that emergency myelopoiesis occurs in severe COVID-19. Silvin et al*.* showed massive amounts of calprotectin and immature myeloid precursors raising the hypothesis of a probable expansion of myeloid-derived suppressor cells during SARS-Cov-2 infection [5, 9]. Hereby, we demonstrated the massive presence of M-MDSC in critically ill COVID-19 patients and its association with mortality. Few preliminary data suggest the presence of myeloid-derived suppressor cells and their role in severe COVID-19 and its pathophysiology [28–30]. Bordoni et al*.* demonstrated that patients with severe COVID-19 admitted in ICU had decreased frequency of lymphocyte T and natural killer, which paralleled expansion of MDSC and high level of cytokines [31]. In complement to our results, Sacchi et al*.* showed that polymorphonuclear (PMN)-MDSC percentage > 54% was associated with mortality in severe COVID-19, heralding the importance of MDSC in severe COVID-19 [30]. Although accumulation of immature myeloid cells is a recognized severity marker of sepsis since the early 1970s, the role of MDSC in critically ill septic patient’s pathophysiology has been poorly studied [5, 32]. Myeloid-derived suppressor cells are bone marrow heterogeneous myeloid immature precursors that may in certain pathological conditions, such as cancer, sepsis, and autoimmune diseases, partially interrupt their differentiation and expend. Two types of myeloid-derived suppressor cells are identified whether originating from PMN or monocytes precursors. MDSC induces lymphocytes T and natural killer cells apoptosis, inhibits T cells proliferation, induces expansion of T regulatory cells, and produces immunosuppressive cytokines such as IL-10 [33, 34]. Both high proportion of PMN- and M-MDSC were shown to be associated with development of secondary infections and early mortality [35–38]. Waeckel et al*.* in a series of secondary gated M-MDSC-like cells (CD14^+^HLA-DR^low^, neither CD15 nor CD33 staining) in septic patients, identified a cut-off value of > 9% significantly associated with 28-day mortality and occurrence of secondary infections [35]. Altogether, there is convergent data suggesting that MDSC are central in the pathophysiology of sepsis, but its regulation, especially in regard to the temporal and spatial organization, seems to be essential in severe COVID-19 [9, 27].

Our study has several limitations. Inherent to the design, this study remains a preliminary single-center observation in need of further validation. The limited number of patients in the three clusters and their association with outcome warrant further confirmation. Nevertheless, the phenotypic characterization of the three clusters and their reciprocal differences are clear enough to suggest association with specific clinical courses and may be of some utility to select patients for innovative therapy [21, 25, 39].

To conclude, our data suggest that mHLA-DR kinetics allows to identify three myeloid phenotypes in critically ill COVID-19 adult patients potentially associated with specific infective paths: hyperactivated monocyte/macrophage, transient immunodepression, and persistent immunodepression phenotypes.

## Supplementary Information

**Additional file 1: Figure S1.** Monocyte HLA-DR variation in patients with or without secondary infection. **Table S1.** Circulating cells phenotyping antibodies clones. **Table S2.** Site of secondary infection and associated pathogens.

## Data Availability

All materials are available upon request to Pr. Pierre Tissières at pierre.tissieres@aphp.fr.

## Abbreviations

COVID-19: Coronavirus disease 2019
mHLA-DR: Monocyte human leukocyte antigen-DR
HAMM: Hyperactivated monocytes/macrophage
PID: Prolonged immunodepression
TID: Transient immunodepression
SARS-CoV-2: Respiratory distress syndrome due to coronavirus 2
CI: Confidence interval
ICU: Intensive care unit
IL−: Interleukin−
TLR: Toll like Receptor
M-/PMN-MDSC: Monocyte-/polymorphonuclear-myeloid-derived suppressor cell
IRB: Institutional board review
EDTA: Ethylenediaminetetraacetic acid
CD: Cluster of differentiation
NK: Natural killer
IDSA: Infection Disease Society of America
AUROC: Area under the receiver operating characteristics
AB/C: AntiBody per cell
ECMO: Extracorporeal membrane oxygenation
