# Supplementary material for: Myeloid phenotypes in severe COVID-19 predict secondary infection and mortality: a pilot study
Source: Ann Intensive Care. 2021 Jul 14;11:111. doi: 10.1186/s13613-021-00896-4 (PMC8278374; doi:10.1186/s13613-021-00896-4)
Supplement: Supplementary file 1 — Additional file 1: Figure S1. Monocyte HLA-DR variation in patients with or without secondary infection. Table S1. Circulating cells phenotyping antibodies clones. Table S2. Site of secondary infection and associated pathogens. [file 13613_2021_896_MOESM1_ESM.docx]

**Myeloid signature in severe COVID-19 predicts secondary infection and mortality. A pilot study.**

Clémence Marais ^1,2^, Caroline Claude ^1,2^, Nada Semaan ^1,2^, Ramy Charbel ^1^, Simon Barreault ^1,2^, Brendan Travert ^1,3^, Jean-Eudes Piloquet ^1,3^, Zoé Demailly ^4^, Luc Morin ^1^, Zied Merchaoui ^1^, Jean-Louis Teboul ^4^, Philippe Durand ^1^, Jordi Miatello ^1,2^ and Pierre Tissières ^1,2^

^1^ Pediatric « adult COVID-19 » Intensive Care, AP-HP Paris Saclay University, Bicêtre Hospital, Le Kremlin-Bicêtre, France.

^2^ Institute of Integrative Biology of the Cell, CNRS, CEA, Univ. Paris Saclay, Gif-sur-Yvette, France.

^3^ Pediatric Intensive Care, Nantes University Hsopital, Nantes, France

^4^ Medical Intensive Care, AP-HP Paris Saclay University, Bicêtre Hospital, Le Kremlin-Bicêtre, France.

**Additional file 1**

**Table S1. Circulating cells phenotyping antibodies clones**

| Circulating cell subset | Marker | Clone | Company |
| --- | --- | --- | --- |
| mHLA-DR | Anti-HLA-DR/Anti-Monocyte Quantibrite assay | L243/ MφP9 | BD Biosciences, San Jose, USA |
|  | CD19 Pacific Blue | SJ25-C1 | Life technologies, Frederick, USA |
| M-MDSC | CD14 PE-Cy5.5 | TuK4 | Life technologies, Frederick, USA |
|  | CD19 Pacific Blue | SJ25-C1 | Life technologies, Frederick, USA |
|  | CD15 BV605 | W6D3 | BD Biosciences, San Jose, USA |
|  | HLA-DR R-PE | TU36 | Life technologies, Frederick, USA |
|  | CD11b FITC | ICRF44 | Life technologies, Frederick, USA |

Abbreviations: CD, Cluster of Differentiation; HLA-DR, Human Leukocytes Antigen-DR; M-MDSC, myeloid Monocyte Derived Suppressor Cell, PE-Cy5.5, phycoérythrine- Cyanine 5.5s; R-PE, R-Phycoérythrine; FITC, Fluorescéine isothiocyanate

**Table S2. Site of secondary infection and associated pathogens**

| **Patient** | **Site of infection** | **Microbiologically documented** |
| --- | --- | --- |
| Patient 1 | VAP | *Pantea septica and Aspergillus Nigri* |
| Patient 2 | NI | negative |
| Patient 3 | VAP | negative |
| Patient 4* | VAP | *Pseudomonas aeruginosa* |
| Patient 5 | VAP | negative |
| Patient 6 | VAP | *Citrobacter krosei* |
| Patient 7 | VAP | *Pseudomonas aeruginosa* |
| Patient 8 | NI | negative |
| Patient 9 | NI | negative |
| Patient 10 | VAP | *Corynebacterium striatum, Enterobacter aerogenes* |
| Patient 11* | VAP | *Aspergillus Fumigati* |
| Patient 12 | VAP | *Corynebacterium striatum* |

NI, no foci identified

* Pulmonary abcess

**Figure S1. Monocyte HLA-DR variation in patients with or without secondary infection.**

mHLA-DR, monocyte Human Leukocyte Antigen – DR; TP 2-1: Difference between Time period 2 (day 5-7) and period 1 (day 1-4); TP 3-2: Difference between Time period 3 (day8-10) and period 2. ** p<0.05*.

**
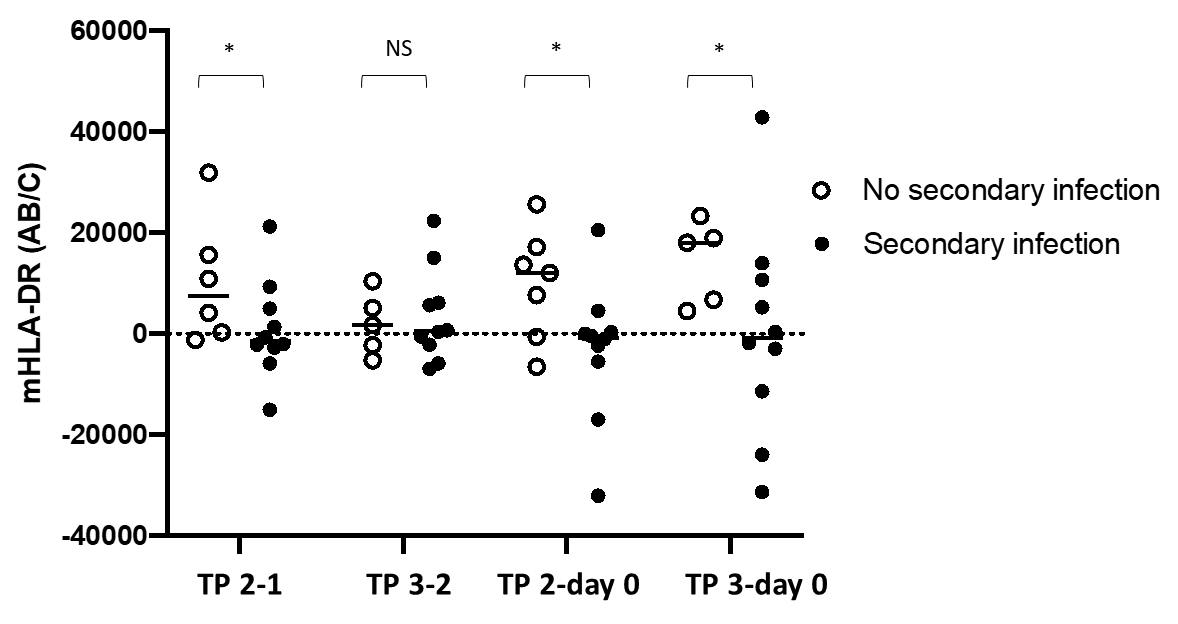
**
